# Outcome of flexible fixation for acute isolated syndesmotic injuries

**DOI:** 10.1186/s12891-024-07849-5

**Published:** 2024-10-03

**Authors:** Hossam El-Azab, Abdel Rhman Hafez, Mohamed A. Mohamed, Moustafa Elsayed

**Affiliations:** 1 Department of Orthopedics and Traumatology, Sohag Faculty of medicine, Sohag University, Sohag, Egypt; 2 Clinic, Behind Luxor International Hospital, Luxor, Egypt

**Keywords:** Acute isolated syndesmotic injuries, Suture–button, Syndesmosis-reconstruction, Syndesmotic ligament

## Abstract

**Background:**

Management of syndesmotic injuries with screw fixation has potential disadvantages, which may lead to the loss of some of the ankle functions. The use of the suture-button system instead can overcome these disadvantages.

**Patients and methods:**

In a prospective study, 32 patients with acute isolated syndesmotic injuries were treated with a suture-button device. Follow-up was for a minimum of 2 years, regarding the Visual Analogue Scale (VAS), American Orthopaedic Foot and Ankle Society (AOFAS) score, patient satisfaction at 3, 12, and 24 months, and radiological assessment.

**Result:**

A significant improvement regarding pain (VAS during rest 5.6 and during walking 6.1 preoperative improved to 0.1 and 0.2 postoperatively, respectively. (*P* values were < 0.0001 for both pain during rest and walking) and AOFAS score (improved significantly from 44 ± 7.5 pre- to 99 ± 8.7 points postoperatively (*P* value was 0.0034). The improved VAS and AOFAS scores of the repaired ankles gradually reached the values of the contralateral uninjured ankle (evaluated at 3,12, and 24 months, postoperatively). Radiographs and CT of both ankles - repaired and healthy ankles - were similar at the 3 months follow-up. Early full weight-bearing and early return to work and sport characterized all patients. There was no need for hardware removal.

**Conclusion:**

Suture-button treatment for acute isolated ankle syndesmotic injuries leads to favorable clinical and radiological outcomes. Postoperative radiographs and CT denoted maintained ankle stability. Patients can do early full weight-bearing and early return to work and sport.

## Introduction

Ankle sprains and fractures are two of the most common injuries associated with sport-related activities [1]. Isolated syndesmotic injuries; without fractures, are more common in athletes when compared to the general population [1, 2].

In ankle sprains, the reported incidence of syndesmotic injuries ranges from 1 to 18%. However, its incidence increases from 13 to 50% when there is an associated ankle fracture, probably because of high-energy ankle trauma. The physician needs a high index of suspicion regarding the mechanism of injury to diagnose a syndesmotic injury [3]. Non-treated syndesmotic ankle instability can evolve into ankle osteoarthrosis [4]. For this reason, the diagnosis and treatment of these injuries are of utmost importance.

Reduction and syndesmotic screw (SS) fixation is the current standard treatment for syndesmotic injuries of the ankle. The screw holds the fibula in the correct position about the tibia while the syndesmotic ligaments heal. However, SS has potential disadvantages [5, 6]. These include screw breakage, loosening, and stiffness of the ankle joint caused by prolonged immobilization. A major disadvantage of screw fixation is that it provides rigid fixation of a joint where physiologic movement should occur.

The syndesmosis allows ankle mortise flexibility due to the elastic nature of the syndesmotic ligaments. Therefore, the intermalleolar space can fluctuate, allowing tibial and fibular rotation with a range of motion of the ankle joint [7], which is impaired in cases of SS. Other disadvantages include the need for a second surgery to remove the screw, hence the risk of developing a late diastasis, if the fixation is removed before the syndesmotic ligaments have completely healed [8]. However, the screw removal is debatable [9].

Suture-button (SB) device is a treatment for syndesmotic injuries that overcomes the disadvantages of the SS. In addition, it is more physiological [10] and allows ankle mortise flexibility and motion. It is unlikely to fatigue and routine removal is not required. Therefore, the patient can start rehabilitation at an earlier stage as the SB substitutes the ligament in load sharing until it has healed.

The objectives of this prospective study were to determine the clinical and radiological outcomes after SB device fixation for acute isolated syndesmotic injuries; without ankle fracture, which is not common.

## Patients and methods

The local ethics committee approved this clinical study. Written consent was collected before study enrolment. From July 2017 to July 2021, forty consecutive patients with acute isolated syndesmotic injuries were enrolled. It was a prospective study for a minimum of 24 follow-up months (range 24–31). There were eight patients lost to follow-up (20%). Finally, 32 patients participated in this study (Table 1).

**Table 1:** Demographic data of patients (N = 32)

Parameter	Patients No.
Patient number	32
Mean age (years)	33.5 (± 9.0)
Male: Female	24: 8
Left ankle: Right ankle	9: 23
Mean interval between injury and operation (days)	9 ± 13
Type of trauma	
Sports	20
Socially	9
Motor car accident	3

The main mechanism of injury was an external rotation force transmitted through dorsiflexion of the ankle. Patients sustained a syndesmotic injury from different traumatic events (Table 1). Acute was defined as **≤** 3 weeks. Isolated was defined as a pure syndesmotic tear not associated with a fracture of the malleoli or a tear of the medial or lateral collateral ligament of the ankle, evaluated clinically and confirmed with imaging. The evaluation was done through a history of the mechanism of trauma, clinical examination (tenderness and hematoma formation over syndesmosis), and confirmation by radiography and ultrasonography.

*The inclusion criteria* involved male and female patients 20 to 50 years of age who presented at our institution with acute isolated distal tibiofibular syndesmotic injuries.

*The exclusion criteria* were patients with syndesmotic injuries associated with a fracture around the ankle joint of the same side or contralateral ankle. Also, cases associated with a deltoid ligament or a lateral collateral ligament injury (cases of Maisonneuve’s fracture acquired MRI of the ankle to check possible associated medial or lateral structure pathology), open or potentially infected ankle fractures, a poly-traumatized patient, osteoarthritis, and the Charcot’s joint were excluded. Patients with neuropsychiatric disorders were also excluded due to low compliance.

From our series of 32 patients, 22 (68.7%) were athletes: {nine Football (28%), three Volleyball (9.3%), five Basketball players (15.6%), and five runners (15.6%). Ten of the athletes played professionally (31.2%).

### Preoperative preparation

Patients were transferred from their location to five different hospitals, which in turn transferred them further to one specific institution. Surgery was performed by the same surgeon.

All patients underwent anteroposterior (AP) and lateral ankle radiographs. Conventional thromboembolic assessment and management was initiated according to national guidelines. Soft tissue swelling was managed with ice compression and elevation. Condition of the soft tissues is the predominant factor that determines the waiting time of surgery, which ranges from 3 to 21 days (9 ± 13 days).

**Fig. 1:**
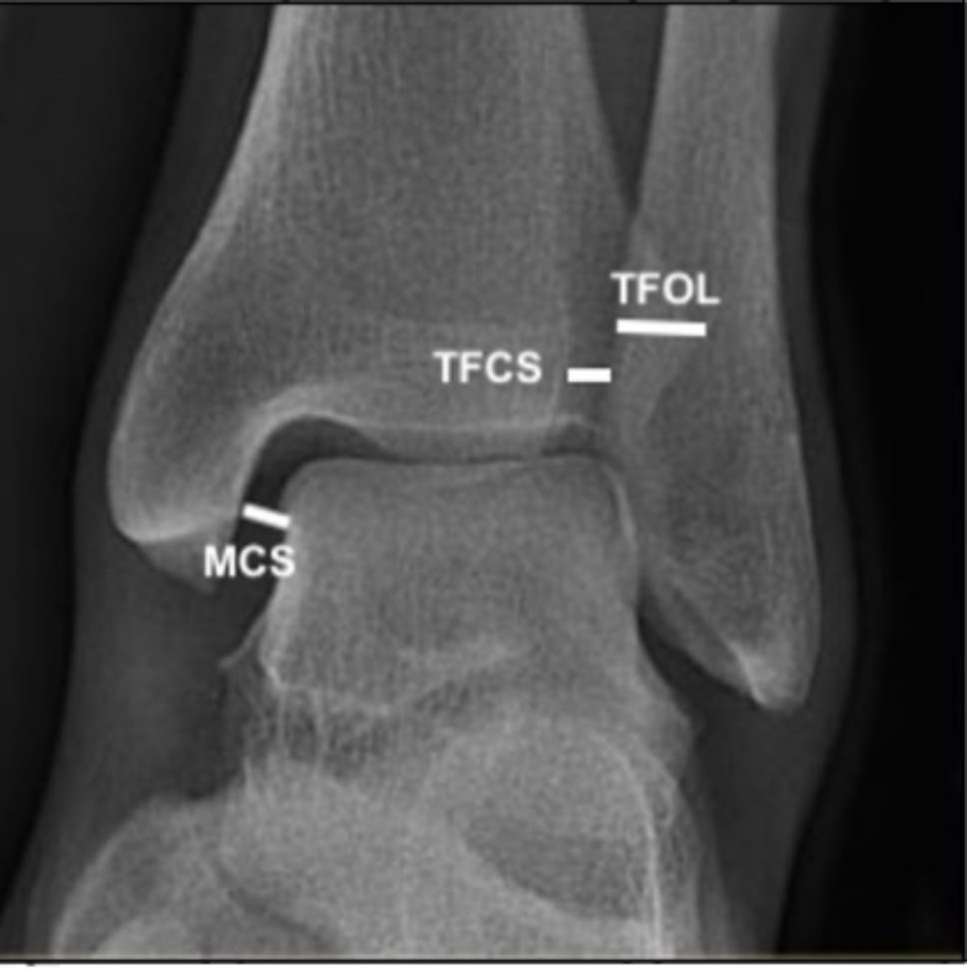
Radiographic AP view of the ankle joint showing the tibiofibular clear space (TFCS), the medial clear space (MCS), and the tibiofibular overlap (TFOL)

AP radiograph was done with the patient in a supine position (Fig. 1; Table 3). The tibiofibular clear space (TFCS) (normal value less than 6 mm on AP radiograph), the medial clear space (MCS) (normal value less than 5 mm on an AP radiograph), and the tibiofibular overlap (TFOL) (normal value more than 6.0 mm on the AP radiograph) [11]. Confirmation was done intraoperatively with fluoroscopy [12].

### Operative technique

The procedure was performed in a supine position on the operating table. At first, lateral traction of the distal fibula horizontally with a hook clamp under the C-arm was performed with a standard 80 ± 11 Newtons traction force to confirm the syndesmotic injury and determine its extent while the foot is fixed by the examiner. The specific method is: one assistant’s hands hold the patient’s knee; the other hand holds the patient’s foot and internally rotates the patient’s tibia 20° (mortise position of the ankle). One of the surgeon’s hands stabilizes the proximal tibia, and the other hand is used to apply traction to the fibula with a bone hook 3–5 cm above the level of the ankle joint. The lateral fibular traction gap is the distance between the fibula and tibia at syndesmosis and was measured on the screen.

According to Qiu et al. [13]. Syndesmotic types were identified by intraoperative fluoroscopy and lateral fibular traction gap on the screen (Table 2); type I, coronal displacement < 4 mm (stable); type II, coronal displacement 4–7 mm (horizontal instability); and type III, coronal displacement > 7 mm (horizontal and vertical instability). Type I was not included in this study [14]. The selection of syndesmosis fixation with the SB device depends on the degree of diastasis. All cases were unstable syndesmotic disruptions of type II or III, according to Qiu et al. classification [13] (Table 2).

**Table 2:** Protocol for syndesmosis injury type and selection of SB fixation, according to Qiu et al. classification [13] (N = 32)

Classification	Hook fibular traction gap	No. of Suture-button	No. of patients
Type I	Syndesmosis gap < 4 mm (stable)	0	0
Type II	Syndesmosis gap 4–7 mm (horizontal instability)	1	12
Type III	Syndesmosis gap > 7 mm (horizontal and vertical instability)	2	20

**Fig. 2:**
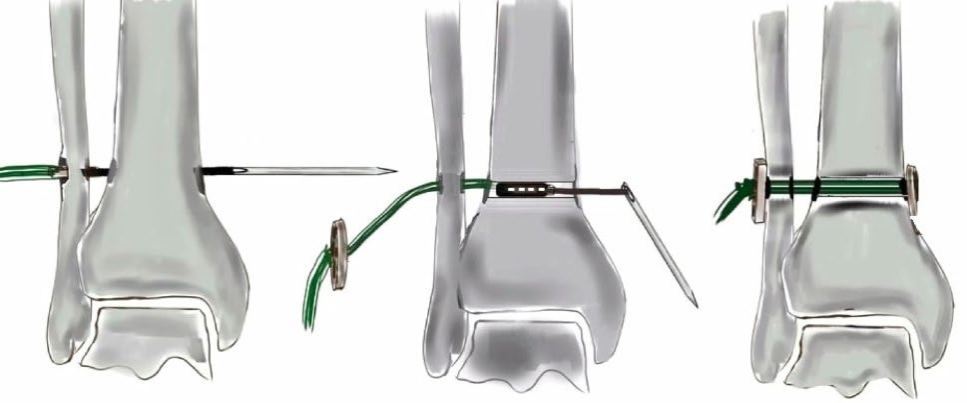
Illustration of the process of suture-button insertion

The technique of SB insertion has already been described before [15] (Fig. 2). Percutaneous drilling of a tibiofibular tunnel with a 4 mm drill bit was done. The tunnel was parallel and 2.5 cm proximal to the joint line in the case of a single SB, or 1.5 cm and 2.5 cm in the case of a double SB. The direction of drilling was from fibula to tibia, divergent in the coronal plane, that is approximately 30° postero-anterior, and in an ascending orientation from posterolateral to anteromedial transversely at an angle of 20–30° with the foot in neutral position [16].

A single device was used for type II injuries. Double SB was performed for type III injuries (Table 3). All steps were performed under fluoroscopy [15] (Fig. 2).

**Fig. 3:**
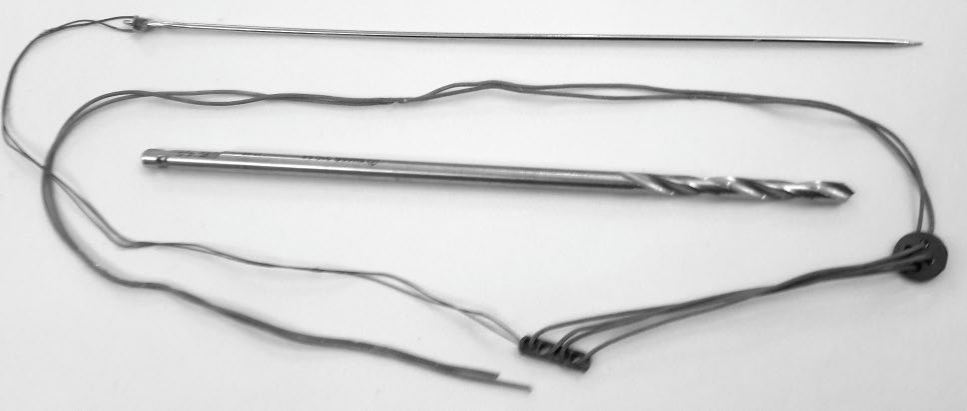
Custem-made Suture-button device; drill bit 4 mm, long straight needle, oblong endobutton, round button and Ethibond No. 5 suture

A custom-made SB assembly was performed preoperatively (Fig. 3). A No. 5 braided polyester suture (Ethibond Excel^®^ Ethicon US.) was looped through the holes of an oblong endo-button and a round button (United Group, Egypt). A pull-through suture, Vicryl No. 0 (Vicryl^®^; Ethicon, Inc. US), was threaded through one of the outer holes of the leading endo-button and a long-straight needle, 15 cm.

**Fig. 4:**
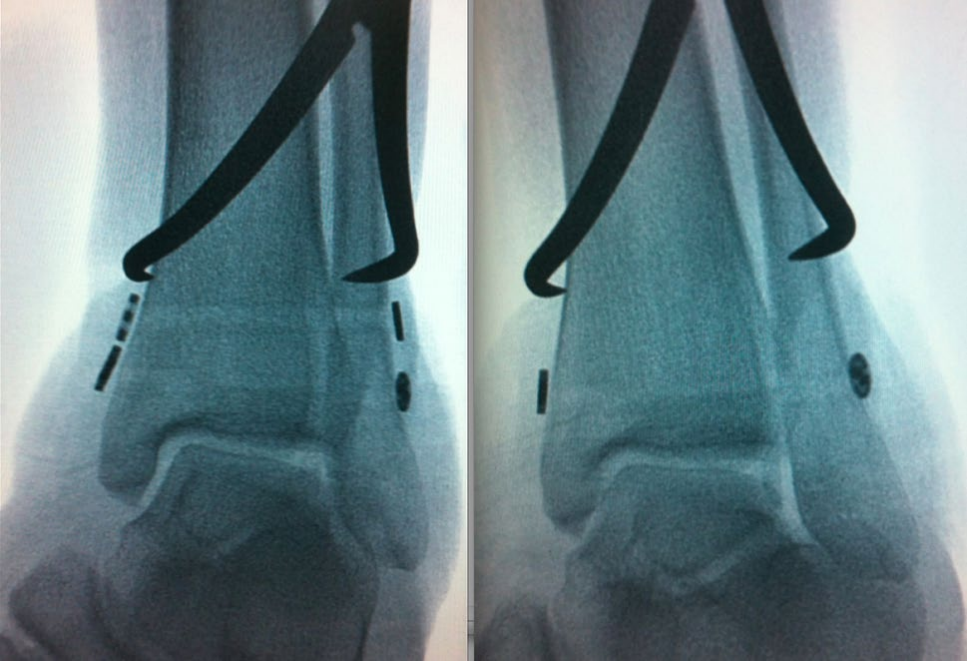
C-arm image showing application of curved reduction clamp either with 2 suture-button (Right) or one suture-button (Left) under reduction

Once the straight needle was retrieved percutaneously at the medial aspect of the tibia and the leading endo-button exited the medial tibial cortex, it was flipped to anchor the medial tibial cortex. Then, utilizing a fixation with a bone clamp, the distance was further narrowed. The trailing round button was tightened by hand onto the lateral side of the fibula and then knotted (Fig. 4).

### Postoperative rehabilitation

All patients were immobilized for the first two weeks in a below-knee plaster slab in neutral flexion, non-weight-bearing. The plaster slab was removed, and patients were instructed to have partial weight-bearing of 20 kg body weight for the following 4 weeks, then full weight-bearing was allowed.

### Outcome measures

**Fig. 5:**
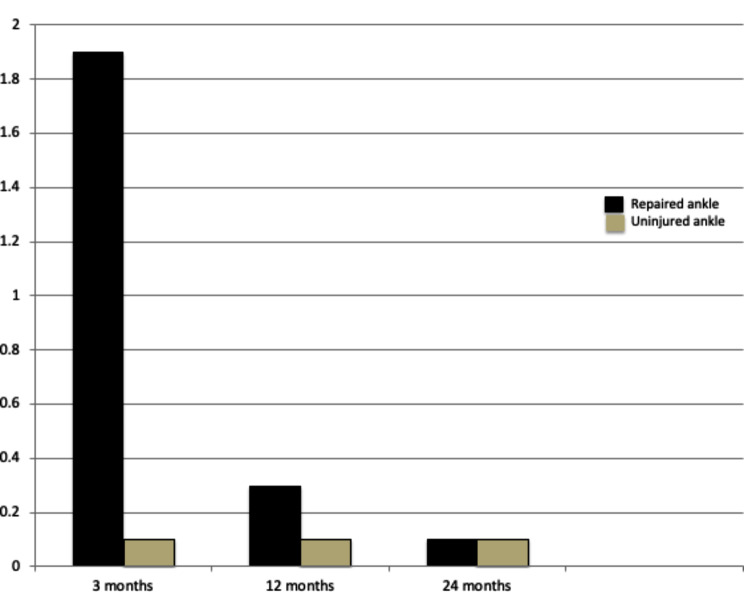
Diagram showing VAS 3,12 and 24 months after surgery. The mean VAS of repaired ankle: healthy ankle were 1.9 (0–3): 0.1 (0–2) 3 months, 0.3 (0–2): 0.1 (0–1) 12 months and 0.1 (0–3): 0.1 (0–1) 24 months, *P* values were 0.1, 0.18 and 0.26, respectively. The number of patients was 32 at 3 months but at 12 and 24 months, the number was 30 patients

**Fig. 6:**
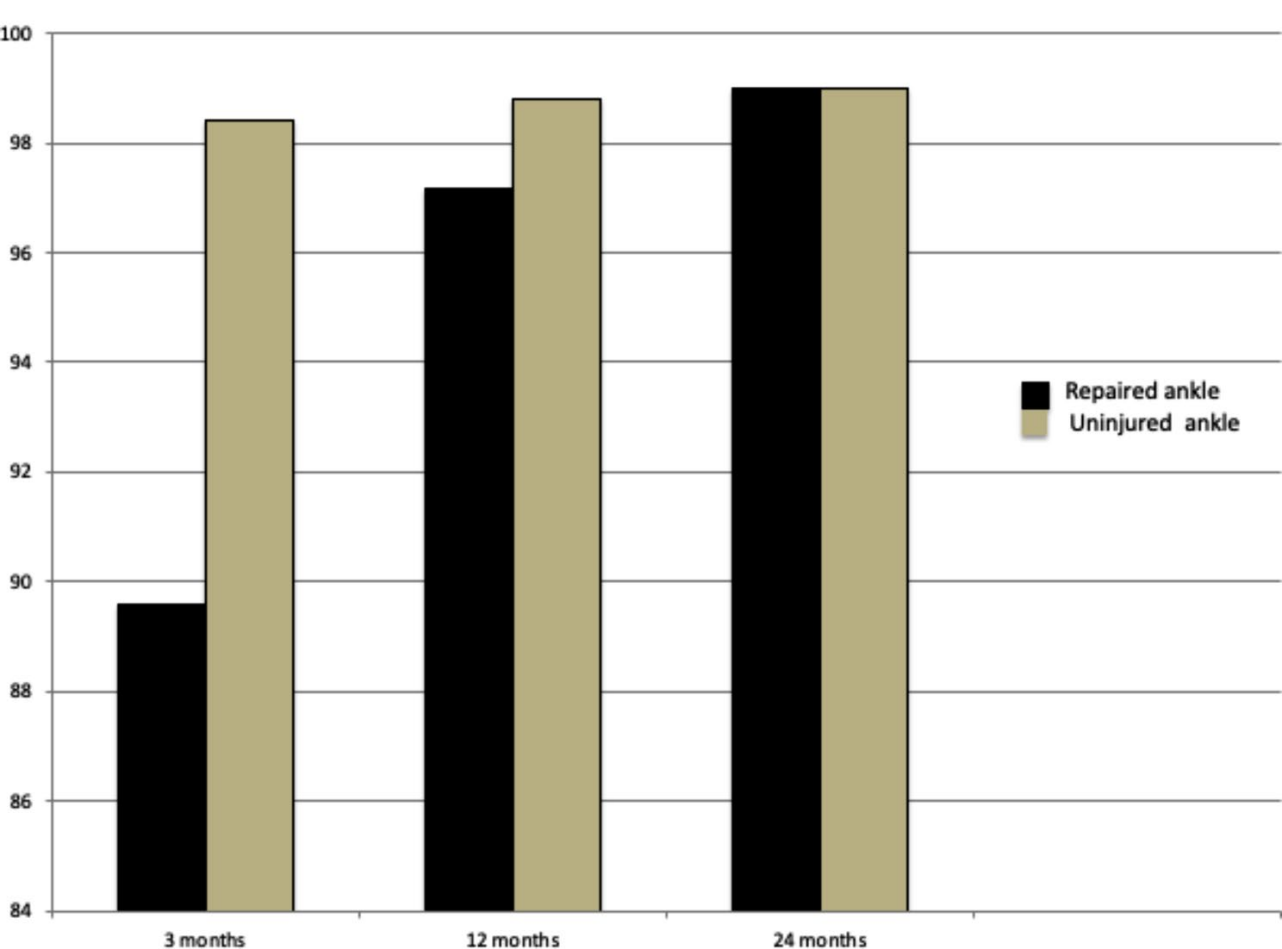
Diagram showing AOFAS score at 3, 12 and 24 months after surgery. The mean AOFAS score of repaired ankle: healthy ankle were 89.6 (86–100): 96.8 (90–100) 3 months, 97.2 (88–100): 98.8 (90–100) 12 months and 99.0 (94–100): 99.8 (97–100) 24 months, *P* values were 0.1, 0.21 and 0.28, respectively. The number of patients was 32 at 3 months but at 12 and 24 months, the number was 30 patients

- *Primary outcome measure*: improvement of clinical and radiological outcomes. The clinical parameters were used to analyze the results: (1) Visual Analogue Scale (VAS) [17], (2) American Orthopaedic Foot and Ankle Society (AOFAS) score [17, 18].(Figures 5 and 6) (3) Patient satisfaction. Radiological assessment using (1) Plain radiographs. (2) Computer tomography (CT).
- *Secondary outcome measure*: Comparison of the repaired ankle with the contralateral, uninjured side 3, 12, and 24 months postoperatively as regard VAS and AOFAS.

**Fig. 7:**
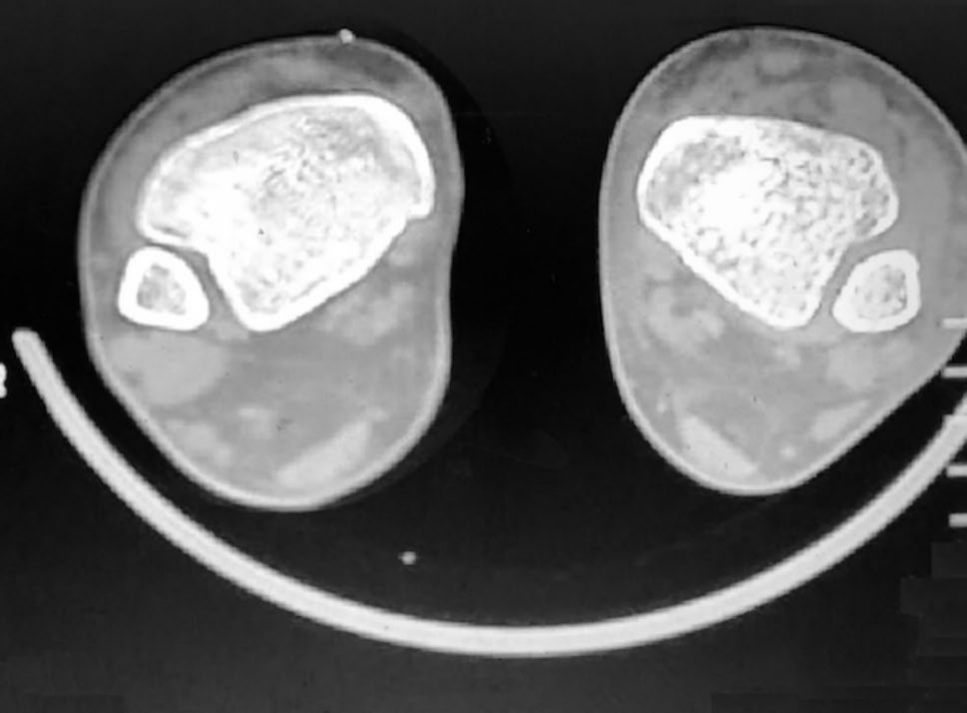
CT 1.5 cm above ankle at both sides at 3 months after surgery. The right side is the operated ankle

Follow-ups sessions were done at two weeks, three months, one year, and two years post-surgery. At the 3 months follow-up, a CT scan was performed 1.5 cm above the ankle joint on both sides. Both feet were loaded during CT, simulating weight-bearing Each foot is pressed against the rigid board during CT. (Fig. 7).

All patients underwent a scoring by a clinician from the research team, who was blinded to the clinical data. Also, radiographs and CT scan evaluations and measurements were done on the computer screen with a special program by a senior radiologist who was blinded to the clinical data. The time to full weight-bearing, return to work, return to sport, or previous sport level were recorded.

### Statistical analysis

The data were analysed using SPSS^®^ software (version 25; SPSS Inc. Chicago, Illinois, USA).Quantitative parametric variables were presented as mean and standard deviation (SD). Paired t- test was used to compare the improvements in the ankle scores pre- and postoperatively after 2 years and to compare between operated and uninjured contralateral ankles. A P-value ≤ 0.05 was considered statistically significant.

A one-way ANOVA test was used to compare between radiological and CT scan measures of affected and nonaffected ankles also to compare between changes in tibiofibular distance over time. A P-value ≤ 0.05 was considered statistically significant (Table 4).

## Results

Clinical evaluation of the operated ankles in two weeks postoperatively showed no pain, and movements were unrestricted without weight-bearing. Full weight-bearing was allowed after six weeks. The mean time to return to work was 2.1 ± 0.7 months. Return to sports was after a mean of 3 ± 7 months. However, the return to the previous preinjury sport level was recorded among athletes to be 5 ± 6 months.

VAS for the patient cohort improved significantly postoperatively. (VAS during rest was 5.6 and VAS during walking was 6.1 preop. improved to 0.1 and 0.2 two years postoperatively, respectively. *P* value was < 0.0001 for both pain during rest and walking) AOFAS score also improved significantly postoperatively (from 44 ± 7.5 pre- to 99 ± 8.7 points), The *P* value was 0.0034. Patient satisfaction for the patient cohort after two years, was excellent in 46% and good in 54%, but there were no fair or poor cases.

Comparisons between the repaired and uninjured contralateral ankles regarding VAS and AOFAS scores at 3, 12, and 24 months postoperatively were done. It showed gradual improvement of the repaired ankle, and reaching the values of a contralateral uninjured ankle after two years. The differences were nonsignificant (Figs. 5 and 6). Also, comparison of ankle measurements on a radiograph after two weeks between the operated, and non-operated ankle was insignificantly different. (Table 3).

**Table 3:** AP-radiographic measurements preoperatively, after 2 weeks post-operatively in the operated ankles and in the uninjured ankles (N = 32)

	Operated ankles preop.	Operated ankles 2 weeks postop.	Uninjured contralateral ankle	P-Value
Mean tibiofibular clear space (TFCS)	7.7 (± 2.5) mm	4.25 (± 4.6) mm	3.7 (± 4.8) mm	0.14
Mean medial clear space (MCS)	4.2 (± 4.4) mm	3.0 (± 3.8) mm	2.6 (± 3.2) mm	0.16
Mean tibiofibular overlap (TFOL)	0.4 (± 0.9) mm	6.84 (± 6.4) mm	7.82 (± 7.6) mm	0.32

*P*-value for the difference between operated and uninjured contralateral ankles two weeks postoperatively

Radiological evaluations of the ankles were scheduled for two weeks, three months, one year, and two years. The tibiofibular distance (distance between the two buttons in the AP radiographs) at different periods postoperatively (Table 4), showed minimal changes. The one-way ANOVA test was 0.783, nonsignificant.

**Table 4:** Mean tibio-fibular distances between the 2 buttons in AP radiographs in two weeks, three months, one and two years (no. Was 32 in 2 weeks, 3 months. However, no. Was 30 in 1 and 2 years). The changes were very small

Mean tibio-fibular distance (between the two Endobuttons)	Postoperative duration of radiographs
49.2 mm.	2 weeks
48.8 mm.	3 months
47.6 mm.	One year
47.2 mm.	Two years

In a comparison between the repaired and uninjured ankle, CT scan 3 months postoperatively (Fig. 7). The mean tibiofibular distance on CT in the repaired ankles was 3.8 ± 0.6 mm, compared to 3.2 ± 0.4 mm on the uninjured contralateral ankle. These were statistically nonsignificant differences (*P* value 0.177).

The athletic patients (No. 22) were monitored very closely, weekly, during their return to sport to guide them. The athletic patients, including professionals, returned to their sport after a mean of 12 ± 7 weeks (or 3 ± 0.3 months). All ten professional athletic patients returned to their pre-injury sport level at different time intervals, with a mean of 5 ± 0.7 months.

There were five Maisonneuve fractures (15.6%), which harbored fracture of proximal fibula and complete separation of syndesmosis; belonged to type III syndesmotic injuries and appropriate for SB treatment as they had a pure ligamentous ankle injury at the ankle. The MRI of those five patients confirmed no accompanying deltoid ligament or lateral collateral injury.

There were no major complications. Three patients (7%) developed local infections at the entry site and responded adequately to antibiotics. Two patients (4.7%) developed irritation at the button site, both medially and laterally. They were removed four months postoperatively. Those two patients were excluded at a later follow-up of the study to avoid bias. They had adequate conditions of ankle mortise and acquired no further surgery.

## Discussion

The most important findings of this study were the significant improvement in clinical and radiological outcomes with SB treatment and the efficient reduction of syndesmosis that was maintained over the follow-up period with early recovery of activity; sports and work. Early and rapid rehabilitation after SB treatment is extremely important for sports players to resume activity and for workers to minimize the days off from work. Comparing the operated ankle with the uninjured contralateral one, the operated ankle gradually improved in functional ankle scores, approaching those of the uninjured ankle.

Pain decreased significantly postoperatively; during rest and walking (*P* value < 0.0001 for both during rest and walking). Which agrees with the comparative study of Andersen et al. [9] between the SB and SS, who reported less pain during rest (*P* = 0.04) and walking for the SB than SS group (*P* = 0.008) after two years of follow-up.

AOFAS score improved significantly postoperatively *(P* value = 0.0034). Similarly, Imam et al. [19] found the AOFAS score improved significantly 24 months postoperatively for SB (*p* < 0.0043).

The mean VAS and AOFAS scores of the operated ankle gradually improved over time in comparison to the healthy ankle (Figs. 5 and 6). The differences between both ankles in VAS and AOFAS scores in all follow-up periods were nonsignificant (*p* values > 0.05).

Patients’ overall satisfaction ratings at 24 months postoperatively showed excellent (46%) and good (54%) results, with no fair or poor results. This is comparable to Thornes et al. [8], who showed excellent, good, and fair satisfaction in the SB group. However, there were greater number of fair or poor results with SS fixation.

Avoidance of late diastasis is ensured by the SB device, which harbours a nonabsorbable braided polyester suture that retains its strength and integrity while the injured syndesmotic ligaments heal [20].

Radiological evaluations of all patients over the two years showed minimal changes in tibiofibular distance between the two buttons. Therefore, there was no loss of tension (Table 4). Kimura et al. [21] similarly found with SB a minimally detectable change in the tibiofibular distance over time. However, Peterson et al. [22] with SB found that, the distance between the buttons increased on average by 1.1 mm from immediate postoperative to final follow-up at two years, demonstrating a loss of fixation.

In the current study, loaded CT scans 3 months postoperatively showed that, the mean tibiofibular distance in the repaired ankles was 3.8 ± 0.6 mm compared to 3.2 ± 0.4 mm in the uninjured contralateral ankle. These were statistically nonsignificant differences (*P* value = 0.177) (Fig. 7). Therefore, there was no loss of tension or diastasis. These findings agree with the comparative study of Thornes et al. [8], who found similar results in CT scans for the SB group. However, Anderson et al. [9] in their comparative study found that in CT, the tibiofibular distance changed in 20% of patients by ≥ 2 mm between the injured and uninjured ankles in the SB group at two-year follow-up (*P* = 0.009).

All patients in this study presented full weight-bearing 6 weeks after surgery and an early return to work after a mean of 2.1 ± 0.7 months. In contrast to Thornes et al. [8], they demonstrated a return to work of an average of 2.8 months for the SB group and 4.6 months for the SS group.

In the current study, the athletic patients, including professionals, returned to their sports after a mean of 12 ± 7 weeks. However, the professional athletes returned to their pre-injury sport level in 20 ± 4 weeks. This was comparable to Colcuc et al. [23] in their comparative study, who found the time required to return to sports activities was significantly shorter in the SB group compared to the SS group (14 versus 19 weeks).

In the present study, the use of a second SB was justified in highly unstable distal tibiofibular joint injuries (type III syndesmotic injuries). This agrees with Naqvi et al. [24] for the use of an extra-SB implant to gain accurate syndesmosis reduction.

In the current study, no suture-button removal was planned. However, they were removed in two patients (6.2%) because of local irritation. Zhang [25] reported implant removal in 5 of 134 (3.7%) patients treated with the SB device.

SB fixation may be particularly advantageous in low bone quality or osteoporotic bones, where screw stability can be impaired [26].

Cadaveric and biomechanical studies have demonstrated that the use of the SB technique provides similar biomechanical strength as SS [27]. Also, clinically, Xu et al. [28] concluded that the SB fixation shows equivalent efficacy to the traditional SS.

There is a cost-benefit of using SB fixation; as the cost of a second operation for implant removal is eliminated [29], and an earlier return to work is beneficial to the patient and the economy. These benefits would compensate for the implant cost, in addition to the problems encountered with SS [30].

However, there are some disadvantages to this SB technique. Imam et al. [31] described in their series one failed SB device for an isolated syndesmotic injury due to imperfect tightening of the threads. Storey et al. [32] determined skin irritation by SB, re-diastasis or failed stabilization, loosening due to osteolytic reaction, and osteomyelitis surrounding the implant.

The suture-button system provides favourable clinical and radiological outcomes for isolated acute syndesmotic injuries. It permits early-weight-bearing and returns to work or sports with the maintenance of syndesmosis reduction.

Innovative in this study; it included patients with isolated syndesmotic injuries, while the literature reported syndesmotic injuries associated with ankle fractures. Also, the relatively large number. As most hospitals reported few cases over a long period of time. Also, this study compared the injured ankle with the uninjured contralateral one.

The major limitations of this study are the absence of a comparison group because the SS is no longer performed at our institution. Also, the study results could have been influenced by the heterogeneity of patients including athletes and others: clerks, housewives, and students. However, despite these limitations, we believe that the results of this study could be useful in the future development of prospective comparative studies between the methods of treatment.

## Data Availability

Data availability The data sets used and/or analyzed during the current study are available from the corresponding author on request.
